# Ms1, a novel sRNA interacting with the RNA polymerase core in mycobacteria

**DOI:** 10.1093/nar/gku793

**Published:** 2014-09-12

**Authors:** Jarmila Hnilicová, Jitka Jirát Matějčková, Michaela Šiková, Jiří Pospíšil, Petr Halada, Josef Pánek, Libor Krásný

**Affiliations:** 1 Department of Molecular Genetics of Bacteria, Institute of Microbiology, Academy of Sciences of the Czech Republic, Prague 142 20, Czech Republic; 2 Department of Molecular Structure Characterization, Institute of Microbiology, Academy of Sciences of the Czech Republic, Prague 142 20, Czech Republic; 3 Department of Bioinformatics, Institute of Microbiology, Academy of Sciences of the Czech Republic, Prague 142 20, Czech Republic

## Abstract

Small RNAs (sRNAs) are molecules essential for a number of regulatory processes in the bacterial cell. Here we characterize Ms1, a sRNA that is highly expressed in *Mycobacterium smegmatis* during stationary phase of growth. By glycerol gradient ultracentrifugation, RNA binding assay, and RNA co-immunoprecipitation, we show that Ms1 interacts with the RNA polymerase (RNAP) core that is free of the primary sigma factor (*σ*^A^) or any other *σ* factor. This contrasts with the situation in most other species where it is 6S RNA that interacts with RNAP and this interaction requires the presence of *σ*^A^. The difference in the interaction of the two types of sRNAs (Ms1 or 6S RNA) with RNAP possibly reflects the difference in the composition of the transcriptional machinery between mycobacteria and other species. Unlike *Escherichia coli*, stationary phase *M. smegmatis* cells contain relatively few RNAP molecules in complex with *σ*^A^. Thus, Ms1 represents a novel type of small RNAs interacting with RNAP.

## INTRODUCTION

Mycobacteria are an important group of bacteria that include lethal human pathogens-*Mycobacterium tuberculosis* and *Mycobacterium leprae*-and nonpathogenic saprophytic species, such as *Mycobacterium smegmatis*. Approximately one third of the world's population is infected by *M. tuberculosis* and almost 9 million people developed the active disease in 2012. According to WHO, tuberculosis caused 1.3 million deaths in 2012. In addition, multi-drug resistant tuberculosis is present in most countries surveyed. An important feature of *M. tuberculosis* is that the bacteria can survive in the human body for decades as a latent infection with no symptoms (1).

Mycobacteria as well as other bacteria need to quickly adapt to changing conditions to survive. When the surrounding environment is favorable, bacteria grow exponentially; in harsh conditions, bacteria enter stationary phase of growth and wait for better conditions. Successful adaptation depends on changes in gene expression. A key molecule participating in this process is RNA polymerase (RNAP) that is itself regulated by various auxiliary factors. Bacterial RNAP is a multisubunit enzyme composed of core subunits: α_2_ββ′ω. The RNAP core associates with different *σ* factors that recognize different promoter sequences, and switching between these *σ* factors regulates gene expression. The number of *σ* factors varies among bacterial species-e.g. *M. smegmatis* has 26 *σ* factors (2,3) while *Escherichia coli* has seven (3). Typically, bacteria have one primary (housekeeping) *σ* factor responsible for the majority of gene expression. This primary *σ* factor is called *σ*^70^ in *E. coli* or *σ*^A^ in *M. smegmatis* and *Bacillus subtilis* (4). When conditions become unfavorable and bacteria enter stationary phase, the transcription of *σ*^70^/*σ*^A^-dependent genes is reduced and genes recognized by alternative stress *σ* factors are activated (5).

Gene expression is also regulated by small RNAs (sRNAs). sRNAs usually have a length of 50–300 nt and most of them base-pair with mRNA and regulate mRNA stability or the efficiency of mRNA translation (6). It is estimated that a bacterial cell such as *E. coli* encodes hundreds of different sRNAs (7). Only a limited number of studies have mapped sRNAs and addressed their function in mycobacteria (8–14). In exponentially growing *M. tuberculosis*, 17% of total non-rRNA transcripts originate from intergenic regions and represent sRNAs (15). This number increases to almost 60% in stationary phase cells, mainly due to the accumulation of a highly abundant sRNA designated MTS2823 (or ncRv13661 according to the new nomenclature (16)). MTS2823 is present in stationary phase cells in amounts comparable to rRNAs (8). An even higher accumulation of MTS2823 sRNA was observed in mice during chronic infection. When MTS2823 was artificially overexpressed in exponential phase cells, the transcription of ∼300 genes decreased (8). Although MTS2823 is highly expressed, affects mycobacterial gene expression and has a possible role during infection, the mechanism of its function is unknown. We previously independently identified a homolog of MTS2823, Ms1, a small RNA in *M. smegmatis* that is highly abundant in stationary phase (17).

Ms1 was originally identified by *in silico* searches for 6S RNA homologs. 6S RNAs fold into a secondary structure that mimics an open promoter (13,18–20) and this structure binds to RNA polymerase in complex with the primary *σ* factor (RNA polymerase holoenzyme). 6S RNA prevents the binding of the RNAP holoenzyme to promoter sequences and reduces its transcriptional activity (21–24). We had originally hypothesized that Ms1 may be the mycobacterial 6S RNA. However, we showed that Ms1 does not interact with the RNAP complex containing the primary *σ* factor (17). In addition, Ms1 has a length of ∼300 nt, while 97% of >3500 known 6S RNA sequences (either predicted or validated) have a length in the 150–210 nt range and no known 6S RNA has a length of ∼300 nt (Rfam database, (25)). 6S RNAs have been found in many bacterial species (20,26–28); mycobacteria are an exception. Despite several studies identifying small RNAs in mycobacteria (8–14), it is still unclear whether mycobacteria possess 6S RNA or not.

Here we use *M. smegmatis* as a model organism and search for the binding partner of Ms1. We show that Ms1 is a sRNA that directly interacts with the transcriptional machinery but in a different way than 6S RNA-Ms1 binds core RNA polymerase instead of RNAP holoenzyme. Thus, Ms1 represents a novel class of small RNAs. Finally, we discuss possible reasons why mycobacteria may differ from most other bacterial species in the interaction of RNAP with the sRNA.

## MATERIALS AND METHODS

### Bacterial strains, growth conditions, plasmids

For detailed descriptions of individual strains see List of strains and plasmids in Supplementary Data. *M. smegmatis* mc^2^ 155 and FLAG-tagged RpoB strain (29) (strain name: MR-sspB; kindly provided by D. Schnappinger, Weill Cornell Medical College, New York, USA) were grown at 37°C in Middlebrook 7H9 medium with 0.2% glycerol and 0.05% Tween 80 and harvested in exponential phase (OD_600_ ∼0.5) or 4–6 h after the entry into stationary phase (OD_600_ ∼2.5–3) unless stated otherwise. Transformations of *M. smegmatis* mc^2^ 155 cells were performed by electroporation. When required for selection of transformants, media were supplemented with hygromycin (50 μg/ml) and/or kanamycin (20 μg/ml). Wild-type *E. coli* K12 KW72 (30), kindly provided by Tamas Gaal, University of Wisconsin-Madison, USA) and *B. subtilis* 168 strains were grown in LB medium and the cells were collected in exponential phase (OD_600_ ∼0.5) or 3–4 h after entry into the stationary phase of growth unless stated otherwise. Growth phenotype experiments (Figure 5C) were conducted in a Tecan Infinite 200 Pro reader and growth was monitored for 24 h.

**Figure 1.**
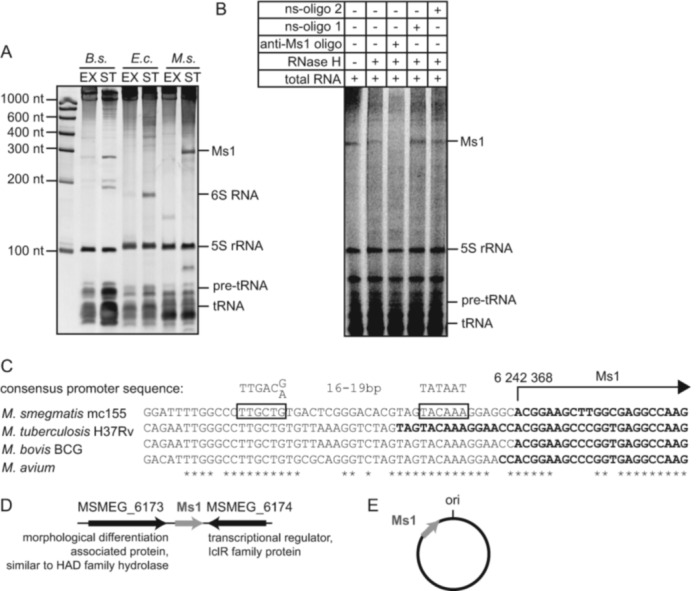
Mycobacterial Ms1 sRNA is expressed in amounts comparable to 6S RNAs. (**A**) Total RNA was isolated from *Bacillus subtilis* (*B.s.*), *Escherichia coli* (*E.c.*) and Mycobacterium *smegmatis* (*M.s.*) in exponential (EX) or stationary (ST) phase. RNAs were resolved on denaturing polyacrylamide gels and stained with GelRed. In *M. smegmatis*, an ∼300 nt sRNA was present in stationary phase cells in amounts comparable to *B. subtilis* or *E. coli* 6S RNAs. (**B**) Before loading onto the gel, total RNA from *M. smegmatis* stationary phase was incubated either with a complementary DNA oligonucleotide (anti-Ms1 oligo) or nonspecific control oligonucleotides (ns-oligo 1 and ns-oligo 2) and treated with RNase H. (**C**) The first nucleotide of Ms1 is adenine transcribed from position 6 242 368 in the genome. The putative −10 and −35 promoter sequences (framed) are perfectly conserved in *M. smegmatis*, *Mycobacterium tuberculosis, Mycobacterium bovis* BCG *and Mycobacterium avium*. The 5′ end sequences of previously identified Ms1 homologs in these species are highlighted in bold. The consensus promoter sequence was adopted from (36). (**D**) The flanking genes of Ms1 in *M. smegmatis* are shown. (**E**) Scheme of Ms1's position in the genome of *M. smegmatis* with respect to the origin of replication (ori).

**Figure 2.**
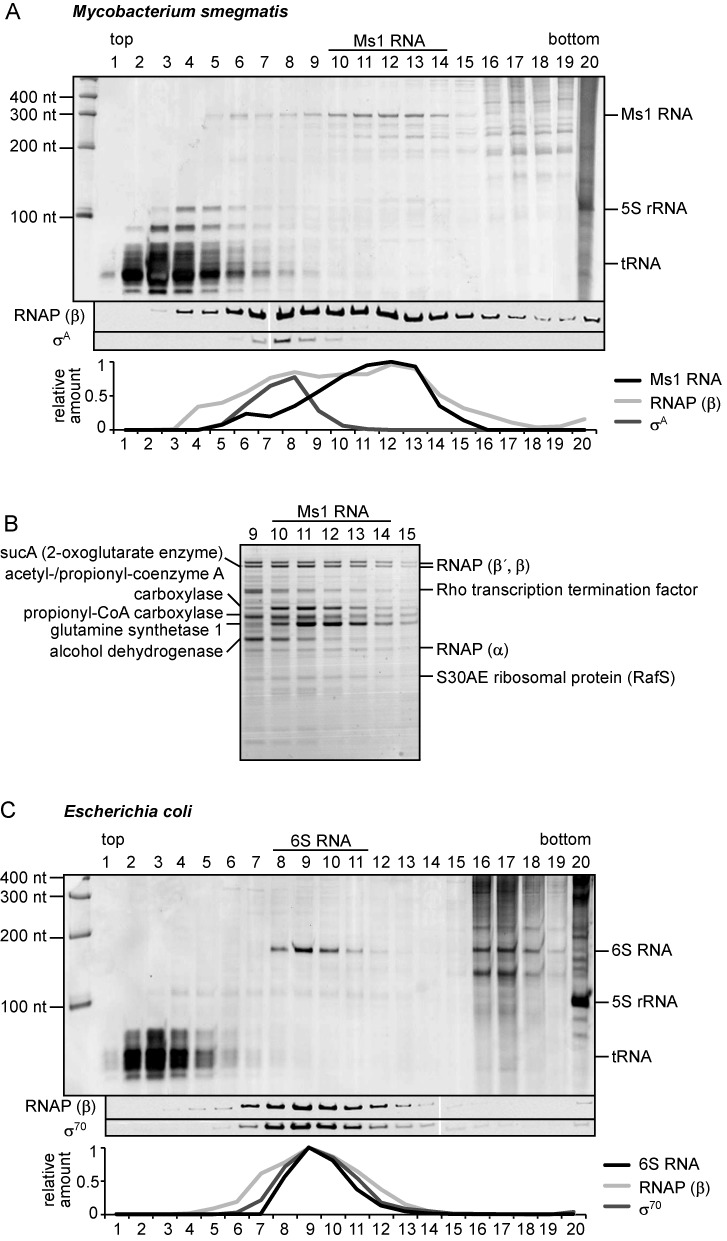
Ms1 is present in a large protein complex. Lysates from stationary phase *Mycobacterium smegmatis* (**A**) or *Escherichia coli* (**C**) cells were fractionated by ultracentrifugation in glycerol gradients; individual fractions (1–20; top to bottom) were collected and the RNAs present in each fraction were resolved on denaturing polyacrylamide gels and stained with GelRed. The RNAP β subunit and primary *σ* factors were visualized by western blotting. Relative amounts of sRNAs (Ms1 and 6S RNA visualized by GelRed) and proteins detected by western blotting are shown below. (**B**) Proteins from Ms1 fractions were separated by SDS-PAGE and stained with Coomassie. RNAP subunits but no *σ* factors were among the most abundant proteins in Ms1 fractions. For details on the mass spectrometry analysis, see Supplementary Table S2. This experiment was performed 3× with identical results.

**Figure 3.**
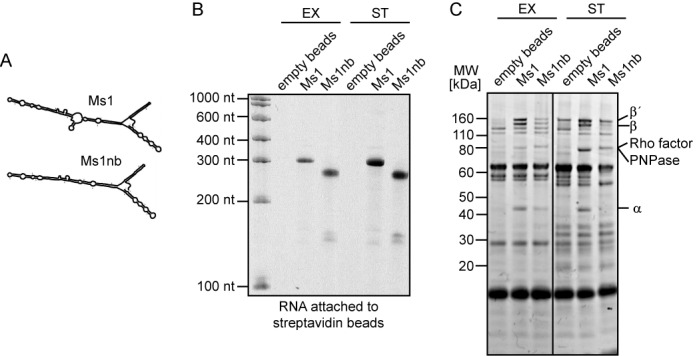
Ms1 pulls down RNA polymerase core. (**A**) Predicted structures of Ms1 and its mutant variant lacking the central bubble, Ms1nb. Both Ms1 and Ms1nb RNAs were prepared *in vitro*, biotinylated and coupled to streptavidin beads. (**B**) One tenth of the RNA-coated streptavidin beads were run on a urea-polyacrylamide gel as a control for the efficiency of biotinylation and coupling. (**C**) The Ms1- and Ms1nb-beads were incubated with *Mycobacterium smegmatis* exponential or stationary phase lysates. Proteins that associated with Ms1 and Ms1nb were separated by SDS-PAGE, stained with Coomassie and Ms1-interacting proteins analyzed by mass spectrometry. Core subunits of RNAP interacted with Ms1 sRNA, but considerably less with Ms1nb and not with empty beads. This experiment was repeated 3× with identical results.

**Figure 4.**
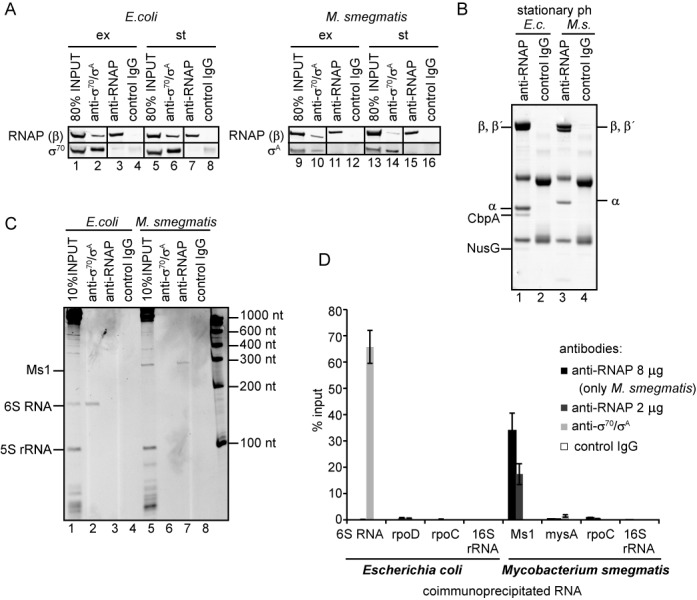
Ms1 interacts with core RNA polymerase. (**A**) Antibodies against RNAP (anti-RNAP; clone name 8RB13-it recognizes the core form of RNAP) and *σ*^A^/*σ*^70^ (anti-*σ*^A^/σ^70^, clone name 2G10-it recognizes also the holoenzyme containing *σ*^A^/*σ*^70^) efficiently immunoprecipitated proteins from both *Mycobacterium smegmatis* and *Escherichia coli.* Immunoprecipitated proteins were visualized by western blotting. ex: exponential phase; st: stationary phase. ‘Control IgG’ is a mouse nonspecific IgG used as a negative control. The experiment was repeated 3× with identical results. (**B**) The RNAP antibody (8RB13) immunoprecipitated the core form of RNA polymerase. Immunoprecipitated proteins from *E. coli* (*E.c.*) and *M. smegmatis* (*M.s.)* stationary phase lysates were separated by SDS-PAGE and stained with Coomassie. ‘Control IgG’ stands for a mouse nonspecific IgG used as a negative control. Additional *E. coli* proteins were identified by mass spectrometry (see Supplementary Table S4). No *σ* factors were detected. No significant bands besides RNAP subunits were found for *M. smegmatis*. (**C**) RNAs coimmunoprecipitated with RNAP (2 μg of antibody) and *σ*^A^/*σ*^70^ antibodies were resolved on PAGE and stained with GelRed or (**D**) quantified by RT-qPCR and normalized to the input. In immunoprecipitations quantified by RT-qPCR, two amounts of the anti-RNAP antibody (8RB13) were used (2 and 8 μg); the amount of coimmunoprecipitated Ms1 increased with the increased amount of the RNAP antibody, suggesting that the concentration of the RNAP antibody was not saturating. *E. coli* 6S RNA coimmunoprecipitated with *σ*^70^ which is in complex with RNA polymerase. None of the control mRNAs-two mRNAs: rpoD (*σ*^70^ mRNA), mysA (*σ*^A^ mRNA), rpoC (RNAP β′ subunit mRNA) and 16S rRNA were coimmunoprecipitated with the antibodies used. ‘Control IgG’ is mouse nonspecific IgG used as a negative control. Error bars are SEM (standard error of the mean) from at least three independent experiments.

**Figure 5.**
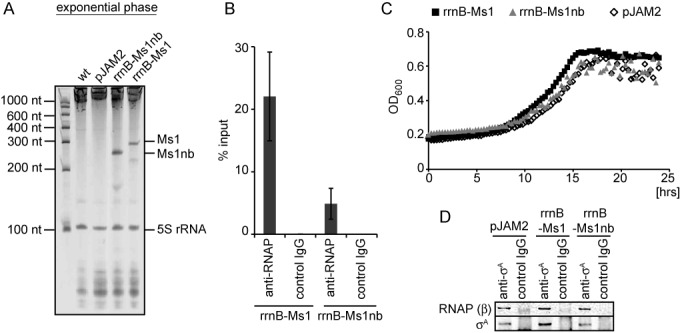
Overexpression of Ms1 in exponential phase. (**A**) Total RNA was isolated from the wt control strain that contained the empty pJAM2 vector and from strains carrying plasmids that contained either Ms1 or Ms1nb under the *rrnB* promoter. The RNAs were then resolved on denaturing polyacrylamide gels and stained with GelRed. Both Ms1 and Ms1nb were highly expressed in exponential phase of growth. (**B**) Core RNAP was immunoprecipitated with 2 μg of the anti-RNAP antibody (8RB13) from strains overexpressing Ms1 or Ms1nb. Coimmunoprecipitated RNA was isolated and the amount of Ms1 or Ms1nb quantified by RT-qPCR. ‘Control IgG’ is a mouse nonspecific IgG used as a negative control. Error bars are SEM from three independent experiments. (**C**) Growth curves of the control strain (pJAM2) and strains overexpressing Ms1 (rrnB-Ms1) or Ms1nb (rrnB-Ms1nb) were compared. The graph shows one representative experiment; the experiment was repeated 3×. (**D**) *σ*^A^ was immunoprecipitated from the control strain (pJAM2) and strains overexpressing Ms1 (rrnB-Ms1) or Ms1nb (rrnB-Ms1nb) and the amount of the coimmunoprecipitated RNAP β subunit was determined by western blotting. No difference in the amount of RNAP-bound *σ*^A^ was detected upon Ms1 or Ms1nb overexpression. ‘Control IgG’ is a mouse nonspecific IgG used as a negative control.

**Figure 6.**
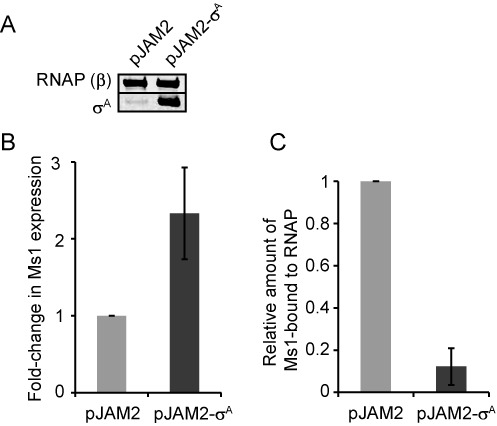
*σ*^A^ overexpression decreases the amount of Ms1-RNAP. (**A**) Protein lysates from *Mycobacterium smegmatis* carrying pJAM2-*σ*^A^ or the empty pJAM2 plasmid were cultured for 6 h in 0.2% acetamide (which induces expression from the pJAM2 acetamidase promoter) and harvested in stationary phase. The amount of *σ*^A^ and RNA polymerase β subunit (RNA polymerase β served as a loading control) was detected by western blotting. Upon induction with acetamide, the *σ*^A^ level increased in *M. smegmatis* stationary phase cells carrying pJAM2-*σ*^A^ but not in cells carrying the empty vector. (**B**) Ms1 sRNA expression increases ∼2.5-fold after the overexpression of *σ*^A^ (Ms1 RNA level was measured by RT-qPCR and normalized to 16S rRNA). (**C**) The amount of Ms1 coimmunoprecipitated with RNAP core decreased more than 8-fold after the overexpression of *σ*^A^ (coimmunoprecipitated Ms1 was first normalized to the input and then to control cells with the empty pJAM2; the graph shows the averages from two independent experiments and the error bars indicate the range).

pJAM2-*σ*^A^ (31) (a gift from V. Nagaraja, Indian Institute of Science, India) contains an inducible acetamidase promoter that allows overexpression of *σ*^A^ upon the addition of acetamide. pJAM2-*σ*^A^ or the empty pJAM2 (32) vector were electroporated into *M. smegmatis* mc^2^ 155, yielding strains LK1304 and LK1302. Acetamide at a final concentration of 0.2% was added to both strains at the entry into stationary phase (OD_600_ = 1.6) and the cells were grown for additional 6 h (experiments shown in Figure 6).

Ms1, Ms1nb (the latter is a mutant Ms1 lacking the internal bubble) and 80 bp *rrnB* promoter (33) DNA sequences (see Supplementary Data) were synthetized by Invitrogen (GeneArt Strings DNA Fragments). By BamHI/KpnI digestion, the acetamidase promoter was deleted from the pJAM2 plasmid and in its place the *rrnB* promoter fused to Ms1 or Ms1nb was cloned using a GeneArt Seamless Cloning and Assembly Kit (Invitrogen; for details see Supplementary Data). Plasmids were verified by sequencing and electroporated into the mc^2^ 155 strain, yielding strains overexpressing either Ms1 (LK1323), or its mutant form Ms1nb (LK1337) or without any overexpression-a control strain with an empty plasmid (LK1302).

### RNA isolation, RNase H treatment and PAGE

The same protocol (adapted from (34)) was used for *E. coli*, *M. smegmatis* and *B. subtilis* RNA isolation. Briefly, 8–20 ml cells from the indicated growth phases were quickly pelleted and immediately frozen. The pellet was then suspended in 240 μl TE (pH 8.0) plus 60 μl lysis buffer (50 mM Tris–HCl pH 8.0, 500 mM LiCl, 50 mM ethylenediaminetetraacetic acid (EDTA) pH 8.0, 5% sodium dodecyl sulphate (SDS)) and 600 μl acidic phenol (pH∼3):chloroform (1:1). Lysates were sonicated in a fume hood, centrifuged, the aqueous phase extracted two more times with acidic phenol (pH∼3): chloroform and precipitated with ethanol. RNA was dissolved in water and DNase treated (TURBO DNA-free Kit, Ambion). Five μg of total RNA were mixed in a 1:1 ratio with the sample buffer (95% formamide, 20 mM EDTA pH 8.0), heated for 1 min at 90°C, then kept on ice and electrophoresed in 7 M urea 7% polyacrylamide gels.

To test whether the prominent 300 nt band in the *M. smegmatis* total RNA consists of Ms1, we used RNase H treatment that degrades RNA in RNA/DNA duplexes. We set up 9 μl reactions with 3.5 μg total RNA, 2 μl 5xRT buffer (from SuperScriptIII CellsDirect cDNA Synthesis System, Invitrogen) and 1 μl 100 μM DNA oligonucleotides (anti-Ms1 oligo 5′-GTCGTGGCCGTCCGCTTTTCGAAACTACGC-3′, ns-oligo_1 5′-CGGGTCACAGCCCAACGTAACTGCCTCAAC-3′ and ns-oligo_2 5′-AAGACTTCGACGTGCGCGACCACCGCAAAC-3′). Reactions were incubated for 1 min at 95°C, 1 min at 60°C and cooled on ice. One μl (2 U) of RNase H (from SuperScriptIII CellsDirect cDNA Synthesis System, Invitrogen) was then added and the reactions were incubated for 10 min at 37°C, mixed 1:1 with sample buffer (95% formamide, 20 mM EDTA pH 8.0), heated for 1 min at 90°C and electrophoresed in 7 M urea 7% polyacrylamide gels. The gels were stained for 20 min with GelRed (LabMark), 10 000× diluted in 1× TBE (Tris-borate-EDTA) gel running buffer and visualized using a UV transilluminator. RNA isolation after the glycerol gradient or immunoprecipitation is described in the relevant sections.

### 5′RACE

Five μg of total RNA was treated with 2 U of Tobacco Acid Pyrophosphatase (TAP; Epicentre) for 1 h at 37°C. RNA was extracted with acidic phenol (pH∼3):chloroform (1:1), precipitated with ethanol and a 5′-adaptor DNA/RNA oligonucleotide (5′-ATCGTaggcaccugaaa-3′, DNA in upper case letters) was ligated to the 5′ ends. RNA was then extracted and reverse transcribed into cDNA (SuperScriptIII, Invitrogen) with an Ms1-specific reverse primer (5′-CGTCCGCTTTTCGAAACTAC-3′). The same reverse primer and the 5′-ATCGTAGGCACCTGAAA-3′ forward primer were used for polymerase chain reaction (PCR) with Taq DNA polymerase (Biotools). The PCR products were sequenced and mapped to the *M. smegmatis* mc^2^ 155 genome (GenBank # NC_008596.1). The transcription start sites for other species were retrieved from previous publications: MTS2823 (TSS 4 100 669, *M. tuberculosis* H37Rv) (8) GenBank #AL123456.2, Mcr8 (TSS 4,073,797, *Mycobacterium bovis* BCG Pasteur 1173P2 genome) (10) GenBank #AM408590.1, igMAV_0468–0469 (TSS 458,799, *Mycobacterium avium* MAH104) (35) GenBank #CP000479.1.

### Glycerol gradient ultracentrifugation and western blotting

*E. coli* and *M. smegmatis* stationary phase cells were pelleted and resuspended in 20 mM Tris–HCl pH 7.9, 150 mM KCl, 1 mM MgCl_2_, 1 mM dithiothreitol (DTT), 0.5 mM phenylmethylsulfonyl fluoride (PMSF) and Calbiochem Protease Inhibitor Cocktail Set III protease inhibitors, sonicated 15 × 10 s with 1 min pauses on ice and centrifuged. Protein extracts (1.5 mg) were loaded on a linear 10–30% glycerol gradient prepared in gradient buffer (20 mM Tris–HCl pH 7.9, 150 mM KCl, 1 mM MgCl_2_, 1 mM DTT, 0.5 mM PMSF and Calbiochem protease inhibitors) and fractionated by centrifugation at 32 000 rpm (130 000 × *g*) for 17 h using an SW-41 rotor (Beckman). The gradient was divided into 20 fractions, RNA from individual fractions was extracted with acidic phenol (pH∼3):chloroform, precipitated by ethanol and electrophoresed in 7 M urea 7% polyacrylamide gels. The gels were stained for 20 min with GelRed (LabMark) 10 000× diluted in 1× TBE gel running buffer and imaged using the UV transilluminator. Proteins were analyzed by sodium dodecylsulphate-polyacrylamide gel electrophoresis (SDS-PAGE) and Coomassie staining (SimplyBlue, Invitrogen) or detected by western blotting using mouse monoclonal antibodies to *σ*^70^/*σ*^A^ [clone name 2G10] or to the β subunit of RNA polymerase [clone name 8RB13] and secondary antibodies conjugated with a fluorophore dye and quantified with an Odyssey reader (LI-COR Biosciences). 6S RNA, Ms1, *σ*^A^, *σ*^70^ and RNA polymerase (β subunit) were quantified from RNA gels/western blots using the ImageJ software.

### Immunoprecipitation and RT-qPCR

*E. coli* and *M. smegmatis* cell lysates were prepared in the same way as the lysates used for the glycerol gradient ultracentrifugation, and 300 μg (protein) of lysates were incubated for 2 h at 4°C with 20 μl of Dynabeads Protein A (Invitrogen) coated either with 4 μg mouse monoclonal antibody to *σ*^70^/*σ*^A^ [clone name 2G10], 2–8 μg mouse monoclonal anti-β subunit of RNAP antibody [clone name 8RB13] (both from Santa Cruz) or 10 μg mouse nonspecific IgG (Sigma-Aldrich) used as a negative control. The captured complexes were washed 4× with 20 mM Tris–HCl pH 7.9, 150 mM KCl, 1 mM MgCl_2_ and divided into two parts. A quarter of the beads were incubated in SDS sample buffer for 5 min at 95°C and eluted proteins were detected by western blotting. The remaining three quarters of the beads were resuspended in 200 μl 1% SDS, 150 mM KCl, 20 mM Tris–HCl pH 7.9, 1 mM MgCl_2_ and vortexed with 200 μl acidic phenol (pH∼3):chloroform (1:1) for 10 min. Eluted RNA was precipitated with ethanol, dissolved in water and DNase treated (TURBO DNA-free Kit, Ambion). RNA was visualized on a 7 M urea 7% polyacrylamide gel by staining with GelRed (LabMark). Alternatively, RNA was reverse transcribed into cDNA (SuperScriptIII, Invitrogen) using random hexamers and amplified by quantitative reverse transcription PCR (RT-qPCR) in a LightCycler 480 System (Roche Applied Science) in duplicate reactions containing LightCycler^®^ 480 SYBR Green I Master and 0.5 μM primers (each). For the Ec_6SRNA primer pair, dimethyl sulfoxide (DMSO) was added to a 1% final concentration to optimize amplification efficiency. Primers were designed with Primer3 software and their sequences are in the Supplementary primer list. Negative controls (no template reactions and reactions with RNA as a template to control for contamination with genomic DNA) were run in each experiment, the quality of the PCR products was determined by dissociation curve analysis and the efficiency of the primers determined by standard curves. The proportions of coimmunoprecipitated RNAs were quantified on the basis of the threshold cycle (Ct) for each PCR product that was normalized to input values according to the formula 2^(Ct(immunoprec)–^^Ct(input))^.

### FLAG-tag pull down

Lysates from MR-sspB (FLAG tag on β) and the wt mc^2^ 155 strain (negative control) were prepared in the same way as the lysates for glycerol gradient centrifugation, 300 μg of lysate incubated with 10 μl MS2 resin (Sigma-Aldrich) for 1 h at 4°C, captured complexes were washed 4× with 20 mM Tris–HCl pH 7.9, 150 mM KCl, 1 mM MgCl_2_ and proteins/RNA isolated and detected in the same way as the immunoprecipitations.

### Biotinylated RNA pull down

Ms1 and Ms1nb templates were amplified from Invitrogen DNA (GeneArt Strings DNA Fragments) with the primer carrying the T7 promoter at their 5′ end (see Supplementary Primer List). RNAs were prepared with a T7 RiboMAX Express Large Scale RNA Production System (Promega) and their 3′ends were biotinylated with an RNA 3′ End Biotinylation Kit (Thermo Scientific). After biotinylation, 2.5 μg of each RNA was denatured for 2 min at 90°C and refolded for 20 min at RT in 200 μl folding buffer (100 mM KCl, 10 mM MgCl_2_, 10 mM Tris–HCl pH 7). RNAs were incubated in the same buffer for 1 h at 4°C with 100 μl magnetic streptavidin-coated beads (Sigma-Aldrich) while being gently agitated. The beads were then washed with lysis buffer (20 mM Tris-HCl pH 7.9, 150 mM KCl, 1 mM MgCl_2_) and incubated with ∼400 μg of cell extract (prepared in the same way as for glycerol gradient ultracentrifugation) for 1 h at 4°C, washed 4× with lysis buffer, resuspended in SDS sample buffer, heated for 5 min at 95°C and the eluted proteins were detected by SDS-PAGE and Coomassie staining (SimplyBlue, Invitrogen).

### Quantification of Ms1 and northern blotting

Cells from stationary phase were used to determine the number of viable cells and purify total RNA. Viable cell numbers were counted as follows: 100 μl aliquots of cells from serial dilutions (10^−5^ to 10^−8^) were plated on Middlebrook 7H10 agar. The plates were incubated at 37°C for 5 days before counting the colony forming units (CFU) and calculating CFU/ml. The obtained data were used in subsequent calculations of amounts of Ms1 per cell. The quantity of Ms1 in the total RNA sample (stained with GelRed) was determined by comparing the Ms1 band density to a standard curve derived from the RiboRuler Low Range RNA Ladder (Thermo Scientific). The quantitation was done with QuantityOne software (Biorad). The Ms1 quantity determined by northern blotting was done by comparing the signal intensity of Ms1 with a standard curve derived from serial dilutions of *in vitro* transcribed Ms1. Northern blotting was performed as described previously (17). Briefly, RNAs were resolved on a 7% polyacrylamide gel and transferred onto an Amersham Hybond-N membrane. Probes were 5′ ^32^P-labeled oligonucleotides (anti-Ms1: see RNA isolation; anti-5S: 5′-CTGGCAGGCTTAGCTTCCGGGTTCGGGATG-3′), and signals were captured in Fuji MS phosphor storage screens and scanned with a Molecular Imager_FX (BIO-RAD) and quantified with QuantityOne software (Biorad). For calculating the increase of Ms1 expression in stationary phase relative to exponential phase, the Ms1 northern blot signal was normalized to 5S rRNA.

## RESULTS

### Ms1 is mycobacterial sRNA expressed in a quantity comparable to 6S RNAs

First, we wanted to explore the relative amounts of Ms1 in *M. smegmatis* and compare it to the amounts of known 6S RNAs in *E. coli* and *B. subtilis* (recently reviewed in (23,24)). 6S RNA in *E. coli* (184 nt) and the main 6S RNA in *B. subtilis* (represented by the 190 and 201 nt doublet) are highly expressed during stationary phase and both are clearly visible when total RNA is resolved on denaturing PAGEs (13,20). We isolated RNAs from *M. smegmatis*, *B. subtilis* and *E. coli* from exponential and stationary phases and resolved them electrophoretically on a PAGE gel (Figure 1A). The gel was stained with GelRed, an intercalating fluorescent nucleic acid gel stain that is more sensitive than ethidium bromide and able to detect nanograms of ssRNA (see Supplementary Figure S1A). By using this total RNA staining we were able to directly compare the amount of individual small RNAs with 5S rRNA. While the 6S RNAs of *E. coli* and *B. subtilis* were prominently visible, no RNA of similar length was detected in *M. smegmatis*. Instead, we observed a ∼300 nt sRNA expressed mainly during stationary phase and its amount was comparable to the quantities of 6S RNAs in *E. coli* and *B. subtilis*. The length of this RNA corresponded to Ms1. To confirm that the ∼300 nt band was indeed Ms1, we incubated total RNA with Ms1-specific or control DNA oligonucleotides and treated the RNA samples with RNAse H that specifically degrades RNA within RNA/DNA duplexes. When incubated with an oligonucleotide complementary to Ms1, RNAse H digested the ∼300 nt band and this band disappeared from the gel; two control oligonucleotides not complementary to the Ms1 sequence did not affect the ∼300 nt sRNA (Figure 1B).

We then quantified the amount of Ms1 in mycobacterial cells using total RNA staining (GelRed, Supplementary Figure S1A) and northern blot hybridization (Supplementary Figure S1B). In both cases we compared the Ms1 amount extracted from a known number of cells to serially diluted standards of known concentration. From the GelRed stained gels, we calculated the amounts of Ms1 and 5S rRNA (as a control) in stationary phase to be ∼400 and ∼2800 molecules per cell, respectively. From the northern blots (Supplementary Figure S1B) we obtained a comparable result of ∼600 Ms1 molecules per cell. Compared to exponential phase, the Ms1 amount increased ∼130-fold upon entry into stationary phase (Supplementary Figure S1B).

Besides Ms1, we also detected a <100 nt band in stationary phase (Figure 1A). Another prominent band of ∼130 nt was detected in exponential phase. The identity of the sRNAs represented by these bands is currently unknown and they are the subjects of another study. Hence, we established that Ms1 is a highly abundant sRNA present in the cell in quantities comparable to the 6S RNA found in other species and we decided to further characterize Ms1.

### Ms1's position on the chromosome, its synteny and secondary structure are conserved in mycobacteria

We began characterizing Ms1 by performing 5′RACE (Rapid Amplification of the 5′ cDNA End) with total RNA isolated from the *M. smegmatis* stationary phase cells and identified the first nucleotide of Ms1 to be an adenine transcribed from position 6 242 368 in the *M. smegmatis* genome (Figure 1C). Upstream of this nucleotide we detected sequences resembling mycobacterial −10 and −35 consensus promoter hexamers for the primary *σ* factor (36). The putative promoter sequence of Ms1 is conserved among *M. smegmatis, M. bovis, M. avium* and *M. tuberculosis* (Figure 1C). Although the transcription start sites of Ms1 homologs in *M. bovis* and *M. avium* (10,35) were mapped previously to the same or nearly identical positions, the 5′terminus of MTS2823, the Ms1 homolog in *M. tuberculosis*, was originally mapped further upstream (see Figure 1C) and no match for the promoter consensus sequence upstream of this nucleotide was reported (8). The strength of the putative Ms1 promoter as well as the identity of the *σ* factor recognizing this promoter need to be further tested, but Ms1 is likely a single gene transcription unit. The putative rho-independent Ms1 terminator was found by TransTermHP (37) and is located 304 nt downstream of the 5′terminus. We identified homologs of Ms1 in many mycobacterial species and also in *Nocardia* and *Rhodococcus* (Supplementary Table S1), which belong to the group of actinobacteria. Based on sequence similarity searches (38), Ms1 was found in 33 actinobacterial species; for the phylogenetic tree (39) of Ms1 homologs see Supplementary Figure S2A. When Ms1 is present, its genomic context (Figure 1D) is partially conserved also in other actinobacterial species (Supplementary Table S1). In addition, Ms1 is very often (in 84% of cases) located at the same position on the chromosome: close to the replication start site (ori) with the direction of transcription toward ori (Figure 1E and Supplementary Table S1). This position is independent of genome size (Supplementary Figure S2B). Next, we aligned all Ms1 homologous sequences and compared their predicted secondary structures (40). *In silico*, we created a ‘common’ structure that is prevalent in all Ms1 homologs (see Supplementary Figure S3). These sequences fold into a long double-stranded hairpin with a bubble in the middle and two short hairpins close to the ends. The internal bubble and the adjacent double stranded regions are the most conserved elements. Although the primary sequences of Ms1 homologs are not identical, their predicted secondary structures are similar, which suggests that these sRNAs could have the same function(s).

### Ms1 is present in a large macromolecular complex

As a first approach to explore whether or not Ms1 is part of a protein complex (analogous to 6S RNA or ribosomal RNAs), we fractionated *M. smegmatis* and *E. coli* (as a control) stationary phase cell lysates by glycerol gradient ultracentrifugation. After ultracentrifugation, large complexes such as ribosomes are sedimented at the bottom of the tube whereas e.g. 6S RNA is found in the middle of the gradient. Importantly, molecules that are in the same complex must be found in the same fractions of the gradient. This method was previously used to identify proteins that are in complex with *E. coli* 6S RNA (21). The majority of Ms1 sedimented in fractions 10–13 (Figure 2A) indicating that Ms1 was part of a large protein complex. Using western blotting, we found the RNAP β subunit in Ms1 fractions; however, no *σ*^A^ was found there (Figure 2A), since *σ*^A^ is not in the complex with Ms1 (17). Using mass spectrometry we identified all abundant proteins present in the Ms1 fractions (Figure 2B and Supplementary Table S2)-the potential interacting partners of Ms1 sRNA. Importantly, the RNA polymerase β, β′ and α subunits, but no *σ*^A^ or other *σ* factors, were among the proteins enriched in the Ms1 fractions. Finally, in contrast to *E. coli* where 6S RNA co-sediments with *σ*^70^ and β (Figure 2C, fractions 8–12), no highly expressed sRNA specifically peaked in the same fractions as the *M. smegmatis* *σ*^A^-RNAP holoenzyme (Figure 2A, fractions 7–8).

### Ms1 interacts with RNA polymerase core devoid of *σ* factors

To identify which of the proteins from the Ms1 fractions truly interacted with Ms1, we pulled down Ms1 binding partners directly. First, we prepared Ms1 sRNA by *in vitro* transcription. In addition, we also prepared Ms1 with a deleted internal bubble-designated ‘Ms1nb’ (Figure 3A). We then ligated biotinylated Cytidine (Bis)phosphate to the 3′ends of both RNAs, and attached these biotinylated RNAs to streptavidin beads (Figure 3B). We subsequently incubated the Ms1 and Ms1nb-coated beads with the lysate from *M. smegmatis* cells from exponential and stationary phases. Ms1 is expressed mainly in stationary phase but we avoided using only stationary phase lysate because it contains abundant endogenous Ms1 that could compete for interacting proteins with biotinylated Ms1 and therefore we might miss some of the interacting proteins. Using this approach, we pulled down significant amounts of the core subunits of RNAP with Ms1, but considerably less with Ms1nb, from both exponential and stationary phases. This indicates that the internal bubble which is structurally conserved among Ms1 homologs is important for the interaction with RNA polymerase. No RNA polymerase interacted with the control beads without RNA (Figure 3C). In addition to core RNAP subunits, Ms1 pulled down a ∼80 kDa band consisting of two proteins according to the mass spectrometry analysis: transcription termination factor Rho and polyribonucleotide nucleotidyltransferase involved in RNA degradation (see Supplementary Table S3). Importantly, no *σ* factor or any other proteins found in the glycerol gradient Ms1 fractions interacted with Ms1, although we cannot exclude the possibility that a highly substochiometric presence of *σ* factors (with respect to the other core subunits of RNAP) might be below our detection limit.

To verify that Ms1 associates with RNAP, we utilized a *M. smegmatis* strain carrying the FLAG-tag on β (RpoB) (29), one of the core subunits of RNAP. Via β we pulled down RNAP (Supplementary Figure S4A) from an *M. smegmatis* stationary phase lysate and isolated RNA associated with RNAP. Interaction of RNAs with FLAG-tagged RNAP was assessed using RT-qPCR, with primers specific for Ms1, as well as 16S rRNA, rpoC and mysA mRNAs as negative controls. While Ms1 interacted with the FLAG-tagged RNAP, we observed no interaction of 16S rRNA, rpoC or mysA mRNAs (Supplementary Figure S4B). This experiment, however, did not enable us to distinguish the RNA polymerase core from the holoenzyme (β is present in both complexes).

To distinguish whether Ms1 binds to the core or holoenzyme (*σ*^A^-containing) form of RNAP we performed immunoprecipitations with two specific antibodies. Both antibodies efficiently immunoprecipitated *E. coli* and *M. smegmatis* proteins (see Figure 4A showing the amounts of pulled-down primary *σ* factors and RNAP core β subunits). The anti-*σ*^A^/*σ*^70^ antibody (2G10) recognized both free *σ*^A^/*σ*^70^ as well as *σ*^A^/*σ*^70^ in complex with RNAP (RNAP holoenzyme). The anti-RNAP antibody (8RB13, anti β) was previously reported to mainly bind the RNAP core devoid of *σ* factors (41,42) and we verified this for stationary phase *M. smegmatis* (Figure 4B, lane 3) and *E. coli* cells (Figure 4B, lane 1). In addition to core RNA polymerase subunits (α, β, β′) we found only two other proteins that immunoprecipitated with the anti-RNAP (8RB13) antibody in *E. coli* (and none in *M. smegmatis*) and identified them by mass spectrometry (see Supplementary Table S4) as NusG (transcription elongation factor) (43) and the DNA-binding factor CbpA (44). Importantly, no *σ* factors were detected either in *M. smegmatis* or in *E. coli* immunoprecipitations. We then isolated the coimmunoprecipitated RNA and visualized this RNA either on PAGE gels (Figure 4C, lane 7) or measured its amount by RT-qPCR (Figure 4D). We found that almost 40% of the Ms1 in stationary cells was bound to mycobacterial core RNA polymerase (Figure 4D, the input represents the total amount of Ms1 isolated from the cell lysate; note that the amount of detected Ms1 depends on the amount of the antibody used). No other small RNAs such as 5S rRNA, tRNAs or the <100 nt and ∼130 nt RNAs identified in Figure 1A were bound to the RNAP core (Figure 4C, lane 7), indicating that the Ms1-RNAP core interaction is specific. Accordingly, three control RNAs (two mRNAs: mysA encoding *σ*^A^, rpoC encoding RNAP subunit β and 16S rRNA) also did not interact with RNAP (Figure 4D). In agreement with previous results ((17) and glycerol gradient ultracentrifugation, Figure 2B), we did not see any binding of Ms1 to *σ*^A^ or the RNAP-*σ*^A^ holoenzyme (Figure 4C, lane 6, Figure 4D). This is in sharp contrast with the situation in *E. coli*, where almost all 6S RNA was pulled down with the antibody against *σ*^70^, as 6S RNA interacts with the RNAP-*σ*^70^ complex (21) (Figure 4C, lane 2, Figure 4D). Furthermore, in *E. coli* no 6S RNA coimmunoprecipitated with the anti-RNAP antibody (Figure 4C, lane 3). Thus, in contrast to 6S RNA, Ms1 interacts specifically with the RNAP core devoid of *σ* factors, representing a novel type of RNAP–sRNA interaction module.

### Ms1 overexpression in exponential phase does not affect *σ*^A^ binding to RNA polymerase

We asked whether Ms1 sRNA overexpression in exponential phase could disrupt the association of the RNAP core with *σ*^A^. To investigate this, we transformed *M. smegmatis* with a plasmid carrying Ms1 under the control of a ribosomal RNA promoter (*rrnB*) that is strongly active in exponential phase (9,33). *In vitro*, Ms1 with a deleted internal bubble (Ms1nb) interacted much less with core RNA polymerase compared to the intact Ms1 (Figure 3C). Therefore we also prepared a strain with Ms1nb under the *rrnB* promoter to test the importance of this predicted structural element *in vivo*. Both RNAs were expressed in high amounts in exponential phase (Figure 5A). Next, we determined whether the artificially overexpressed Ms1 could interact with the RNAP core in exponential phase. Ms1 interacted with the RNAP core in a similar manner to endogenous Ms1 in stationary phase (Figure 5B, ∼20% of total Ms1 present in bacterial cell was pulled down with RNAP when 2 μg of anti-RNAP antibody (8RB13) were used). In addition, <5% of the total Ms1nb associated with RNAP, indicating that the presence of the central bubble increases binding to RNAP. In contrast to the previous observations with a *M. tuberculosis* strain with an overexpressed Ms1 homolog (8), Ms1 overexpression did not slow the growth of *M. smegmatis.* Strains with elevated levels of Ms1 or Ms1nb did not exhibit any significant difference in growth rate from the strain carrying an empty control vector (pJAM2) (Figure 5C). Moreover, the overexpression of Ms1 did not seem to affect the amount of the RNAP β subunit (i.e. the amount of RNAP core) that was bound to *σ*^A^, indicating that the level of RNAP holoenzyme is unchanged despite the elevated Ms1 level (Figure 5D).

### Increased level of *σ*^A^ in stationary phase of growth diminishes the amount of Ms1-RNA polymerase complex

As Ms1 seems to not influence the level of RNAP in complex with *σ*^A^, we asked the reverse question: Can *σ*^A^ affect the amount of Ms1 bound to the RNAP core? To test this *in vivo*, we overexpressed *σ*^A^ in stationary phase cells from the pJAM2-*σ*^A^ plasmid, which contains the *σ*^A^ (*mysA*) gene under an inducible acetamidase promoter (31). As a control, we used cells with an empty pJAM2. Acetamide was added at the entry into stationary phase (OD_600_ ∼1.6) to induce *σ*^A^ expression, cells were harvested after 6 h and the increase in the *σ*^A^ protein level was confirmed by western blotting (Figure 6A). Interestingly, overexpression of *σ*^A^ also increased the expression of Ms1 ∼2.5-fold (Figure 6B), which is in agreement with the presence of the putative *σ*^A^-like promoter sequence upstream of Ms1 in the genome (Figure 1C). We then immunoprecipitated the RNAP core and measured the amount of Ms1 coimmunoprecipitated from cells with pJAM2-*σ*^A^ overexpressing *σ*^A^ and compared it to cells with pJAM2. In cells overexpressing *σ*^A^ we observed an ∼8-fold reduction in the amounts of Ms1 bound to the RNAP core (Figure 6C). Although this reduction can be partially explained by the increase in the total amount of Ms1 in pJAM2-*σ*^A^ cells, the 8-fold decrease in Ms1 bound to core RNA polymerase suggests that *σ*^A^ negatively affects the Ms1–RNAP interaction.

### The amount of RNA polymerase holoenzyme decreases in stationary phase

**Figure 7.**
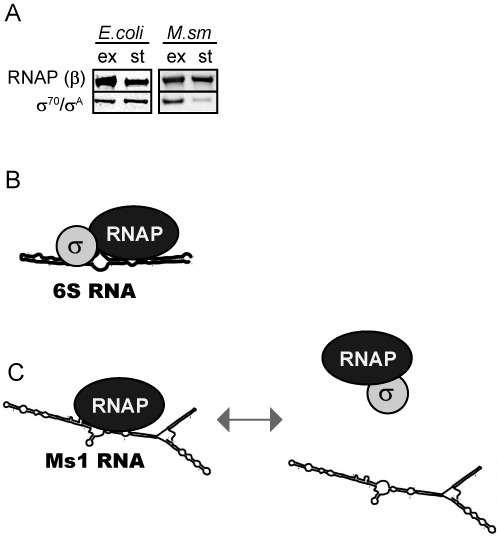
Modes of interaction of sRNAs with bacterial RNAP. (**A**) The level of *σ*^A^ relative to β dropped in *Mycobacterium smegmatis* cells harvested 12 h after entry into stationary phase. In *Escherichia coli*, the relative protein level of *σ*^70^ to β remained unchanged even after 16 h in stationary phase of growth. The experiment was repeated 3× with identical results. (**B**) 6S RNA (e. g. *E. coli, Bacillus subtilis*) binds to RNAP containing the main *σ* factor. (**C**) Ms1 (mycobacteria) binds to the RNAP core in the absence of *σ* factors and the presence of *σ*^A^ decreases this interaction.

Finally, we asked why mycobacterial Ms1 interacts with core RNAP whereas 6S RNA, which is present in the majority of other species, binds to the RNAP holoenzyme. A possible explanation could be that mycobacteria may differ in their amounts of the RNA polymerase holoenzyme in stationary phase. Therefore we compared the amounts of the RNAP core that coimmunoprecipitated with *σ*^70^/*σ*^A^ (the ratio of RNAP to *σ*^A^) in exponential versus stationary phase both in *M. smegmatis* and in *E. coli* by analyzing the data from Figure 4A. While in *E. coli*, a similar amount of RNAP (monitored by the level of β) coimmunoprecipitated with *σ*^70^ both in exponential and stationary phases (compare lanes 2 and 6 in Figure 4A), in *M. smegmatis* fewer RNAPs coimmunopreciptated with *σ*^A^ after the transition from exponential to stationary phase (lanes 10 and 14 in Figure 4A). Thus, in *M. smegmatis* there was a smaller amount of the *σ*^A^-containing holoenzyme in stationary phase when compared to exponential phase, while in *E. coli* the amount of the holoenzyme was about the same. Furthermore, in late stationary phase (12 h into it), the RNAP core had an even lower chance to form a holoenzyme with *σ*^A^ in *M. smegmatis* as we observed a decrease in the total amount of *σ*^A^ in the cells (relative to the RNAP β subunit, Figure 7A) whereas in *E. coli* the total level of *σ*^70^ did not change even 16 h after entering stationary phase (compare the amount of *σ*^70^ to the RNAP β subunit, Figure 7A), suggesting that the decline in *σ*^A^ was specific for *M. smegmatis.* This difference in the *σ*^A^ level in *M. smegmatis* is not visible when the cells are harvested in the early stationary phase (Figure 4A). This means that the adaptation to stationary phase (with respect to *σ*^A^ association with RNAP) in *M. smegmatis* is 2-fold: the binding of *σ*^A^ to RNAP is decreased and the total level of *σ*^A^ drops. Thus, *M. smegmatis* significantly differs from *E. coli* in the composition of the transcription machinery in stationary phase.

## DISCUSSION

### Protein binding partners of Ms1

In this study we characterized Ms1, a highly abundant sRNA from *M. smegmatis*. We determined that Ms1 binds to the RNAP core devoid of *σ* factors. Until now, only 6S RNA was shown to bind to RNAP and the presence of the main *σ* factor is essential for this interaction. Thus, the Ms1–RNAP complex represents a novel type of sRNA–RNAP interaction. Moreover, we detected no other sRNA to interact either with the RNAP core or holoenzyme, suggesting that *M. smegmatis* likely does not contain a 6S RNA. This reflects the difference between mycobacteria and other bacterial species (see ‘The difference between mycobacteria and other bacteria in their interaction of RNAP with sRNAs’ section).

In addition to the RNAP core, two other proteins were found to potentially interact with Ms1. One of them was the transcription termination factor Rho (Figure 3C). It was recently shown that Rho associates with free RNAP as well as with the RNAP/DNA complex (45), which suggests that the Rho factor might be able to associate with the RNAP–Ms1 complex. Future experiments will have to determine whether there is a direct interaction between Ms1 and Rho. Further, Ms1 pulled down polyribonucleotide nucleotidyltransferase (or polynucleotide phosphorylase, PNPase), an enzyme playing both synthetic (as RNA polymerase) and degradative roles (as RNase) in *M. smegmatis* RNA metabolism (46). In *Streptomyces coelicolor*, which belongs to the group of actinobacteria like *M. smegmatis*, PNPase affects the processing of structured RNAs (47). Thus, PNPase could affect the stability of Ms1.

### Ms1 structure

By comparing available homologs, we bioinformatically predicted the secondary structure of Ms1. This secondary structure resembles an open promoter-a long hairpin structure with a single-stranded central bubble. In this respect, Ms1 resembles 6S RNA (13,17,20). Moreover, we showed that the central bubble is important for the interaction with the RNAP core (Figures 3C and 5B). We envisage that Ms1 may interact with RNAP in a similar manner as RNAs of viral origin containing unpaired regions that were shown to be efficiently bound to, and extended by the RNAP core in the absence of *σ* factors (48). Similarly, the RNAP core was reported to bind to artificial DNA templates containing open (bubble) regions without the aid of *σ* factors (49,50). The *σ* factor is not required because the DNA is already open (48–50). The structural details that distinguish Ms1 from 6S RNA will be addressed by future studies.

### Expression of Ms1

The Ms1 level in the cell increases ∼130-fold in stationary phase compared to exponential phase of growth. Although the putative Ms1 promoter sequence resembles a *σ*^A^-dependent promoter, it is not clear which *σ* factor is required for its expression. We observed the accumulation of Ms1 after the overexpression of *σ*^A^ in stationary phase (Figure 6B). However, this still does not prove that the Ms1 promoter is *σ*^A^–dependent and we cannot exclude that the accumulation of Ms1 after the increase in the level of *σ*^A^ in stationary phase is not a secondary effect due to the changed expression of an unknown transcription regulator. An example is the LexA repressor protein that was recently shown to bind at the MTS2823 (Ms1 homolog) locus in the genome of *M. tuberculosis* (51). Other proteins may also associate with the Ms1 genomic locus and participate in the regulation of its expression.

### The quantity of Ms1 in the cell

By two independent methods, we calculated that each stationary phase cell contains several hundred Ms1 molecules (∼400 or ∼600). In stationary phase, almost 40% of total Ms1 sRNA interacts with RNA polymerase, which suggests that ∼200 Ms1 molecules bind around 200 molecules of core RNA polymerase. As a control, we calculated that the number of ribosomes (based on 5S rRNA) is ∼2800 per cell. This is a lower number than what was reported for *E. coli* (52–56) but it likely reflects the presence of only two rRNA operons in *M. smegmatis* (57,58) as well as its relatively slow growth rate (59). The number of RNA polymerase molecules/cell was calculated to be 1400–1500 in slowly growing *E. coli* or *B. subtilis* and the slower the growth rate, the less RNA polymerase was present (53,60–62). In *E. coli*, the majority of RNA polymerase is bound to the DNA and only ∼17% of the molecules are free (63), representing around ∼250 molecules/cell. If similar numbers are applicable to *M. smegmatis*, it would mean that almost all nontranscribing RNA polymerase in the cell is stored in the form of the core enzyme associated with Ms1.

### Connection between Ms1, RNAP core and *σ*^A^

We were able to express Ms1 sRNA in exponential phase in a comparable quantity to the amount of Ms1 present in stationary phase (see Figures 1A and 5A, in both gels the Ms1 sRNA accumulation relative to 5S rRNA was similar). Interestingly, Ms1 expressed in exponential phase interacted with RNA polymerase in comparable amounts to the endogenous Ms1 in the stationary phase (compare % of input in Figures 4C and 5B). This means that ∼200 Ms1 molecules interacted with RNA polymerase in each cell upon Ms1 overexpression. However, cells could efficiently cope with the increase in RNAP-bound to Ms1 because the overexpression of Ms1 did not change the growth rate (Figure 5C). This is in contrast to the situation in *M. tuberculosis*, where overexpression of the Ms1 homolog resulted in a moderate reduction of the growth rate (8). It is possible that under our experimental conditions (*M. smegmatis* grown in rich medium) the cells contain enough free RNA polymerase and thus are not affected by the presence of Ms1, or, there might be different levels of free core RNAP in these two species.

The overexpression of Ms1 in exponential phase did not alter the amount of RNAP holoenzyme containing *σ*^A^. On the other hand, the overexpression of *σ*^A^ in stationary phase decreased the amount of Ms1 bound to RNAP. Taken together, this suggests that Ms1 does not displace *σ*^A^ from RNAP but *σ*^A^ can either prevent the binding of Ms1 to RNAP or help displace Ms1 from the RNAP core. Hence, in the presence of a low concentration of *σ*^A^ Ms1 might stabilize the RNAP core in stationary phase and during dormancy, which is important for the long-term survival of the cell (64). Upon encountering favorable conditions, Ms1 may be released from RNAP and replaced with *σ*^A^ (Figure 7C). Alternatively, by binding to the RNAP core, Ms1 might affect transcription dependent on alternative *σ* factors; this could be important for mycobacteria as they have a higher number of *σ* factors than e.g. *E. coli*.

### The difference between mycobacteria and other bacteria in their interaction of RNAP with sRNAs

We inspected the PAGE gels (Figure 4B) and could not find any RNAs that associated with mycobacterial *σ*^A^ or the RNAP*-σ*^A^ complex in stationary phase (see the empty lane with anti-*σ*^A^). In addition, no sRNA peaks were detected with the RNAP-*σ*^A^ holoenzyme in *M. smegmatis* stationary phase glycerol gradients (Figure 2A, fractions 7–8). This suggests that, in contrast to *E. coli* and many other bacterial species, *M. smegmatis* does not have a 6S RNA that interacts with the RNAP-*σ*^A^ complex. In addition, no abundant sRNA of similar length to 6S RNA (150–210 nt) was present in *M. smegmatis* in either the stationary or exponential phase (in some species 6S RNA can also be expressed in exponential phase (65,66)). Although *M. smegmatis* highly expresses unknown sRNAs <100 nt in stationary phase and ∼130 nt in exponential phase (Figure 1A), we do not assume them to be 6S RNAs because they are relatively short. If *M. smegmatis* possesses any 6S RNA, it is present in significantly smaller amounts than in other species or it must be expressed under specific conditions that we have not tested. Interestingly, 6S RNA was not found in *Corynebacterium glutamicum*, which belongs to the actinobacteria group like *M. smegmatis* (67). Why, then, are mycobacteria different from other bacteria, such as *E. coli* and *B. subtilis*, with respect to the mode of interaction between their respective sRNA and RNAP? The simplest explanation is that they do not need 6S RNA to bind to RNAP-*σ*^A^ because stationary *M. smegmatis* cells contain relatively few RNAP-*σ*^A^ holoenzymes (Figure 4A) and the low level of *σ*^A^ in the cell (Figure 7A) (68). In fact, mycobacteria even need a dedicated RNAP binding protein, RbpA, to stabilize the interaction between RNAP and *σ*^A^ (69). RbpA was shown to be essential in *M. tuberculosis* (70), and until now it has only been found in mycobacteria and other related species within the group of actinobacteria (71,72), indicating a principal difference in the formation and maintenance of the RNAP-*σ*^A^ complex in these species compared to other bacteria. This difference appears to be highlighted by the two types of sRNA interacting in different ways with the transcription machinery (Figure 7B and C). Thus, the regulation of transcription during stationary phase of growth can significantly vary in individual bacterial species and there is no general rule applicable to all bacteria.

## SUPPLEMENTARY DATA

Supplementary Data are available at NAR Online.

SUPPLEMENTARY DATA
