# Supplementary material for: Ms1, a novel sRNA interacting with the RNA polymerase core in mycobacteria
Source: Nucleic Acids Res. 2014 Sep 12;42(18):11763–76. doi: 10.1093/nar/gku793 (PMC4191392; doi:10.1093/nar/gku793)
Supplement: SUPPLEMENTARY DATA [file supp_42_18_11763__index.html]

Ms1, a novel sRNA interacting with the RNA polymerase core in mycobacteria — SUPPLEMENTARY DATA 

# Ms1, a novel sRNA interacting with the RNA polymerase core in mycobacteria

## SUPPLEMENTARY DATA

**Files in this Data Supplement:**

- SUPPLEMENTARY DATA
