# Supplementary material for: Ms1, a novel sRNA interacting with the RNA polymerase core in mycobacteria
Source: Nucleic Acids Res. 2014 Sep 12;42(18):11763–76. doi: 10.1093/nar/gku793 (PMC4191392; doi:10.1093/nar/gku793)
Supplement: SUPPLEMENTARY DATA [file supp_gku793_nar-01445-y-2014-File003.pdf]

Supplementary data

Ms1, a novel sRNA interacting with the RNA polymerase core in mycobacteria

Jarmila Hnilicová[1], Jitka Jirát Matějčková[1], Michaela Šiková[1], Jiří Pospíšil[1], Petr Halada[2], Josef Pánek[3] and Libor Krásný[1,*]

[1] Department of Molecular Genetics of Bacteria, Institute of Microbiology, Academy of Sciences of the Czech Republic, Prague 142 20, Czech Republic,

[2] Department of Molecular Structure Characterization, Institute of Microbiology, Academy of Sciences of the Czech Republic, Prague 142 20, Czech Republic,

[3] Department of Bioinformatics, Institute of Microbiology, Academy of Sciences of the Czech Republic, Prague 142 20, Czech Republic.

* To whom correspondence should be addressed:

Tel: +420 241 063 208, Fax: +420 296 442 201, Email: krasny@biomed.cas.cz

The authors wish it to be known that, in their opinion, the first two authors should be regarded as joint First Authors.

Supplementary Figure 1

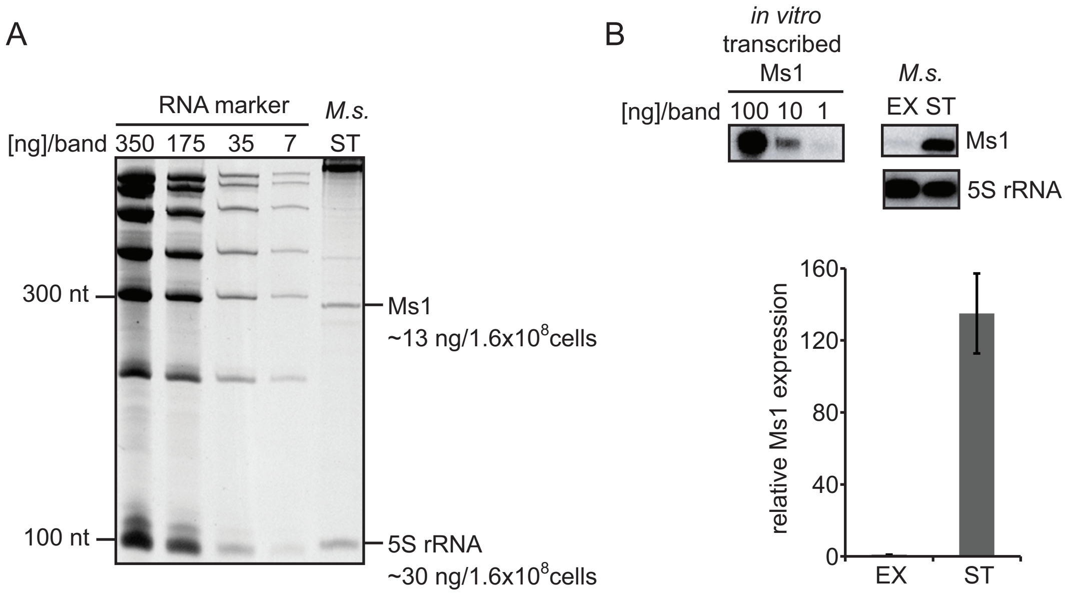

Supplementary Figure 1. Quantification of Ms1 in stationary phase cells. (A) The amount of Ms1 was compared to the 300 nt band of RiboRuler Low Range RNA Ladder (Thermo Scientific) of known concentration. The same approach was used to quantitate 5S rRNA (control). *M.s.* ST is total RNA sample from *M. smegmatis* stationary phase cells. The PAGE was stained with GelRed. **(B)** The amount of Ms1 in total RNA sample from exponential (EX) and stationary (ST) phase was compared to *in vitro* transcribed Ms1 by northern blot analysis. Relative increase of Ms1 in stationary phase is ~130-fold, the graph shows the averages from two independent experiments and the error bars indicate the range.

Supplementary Figure 2

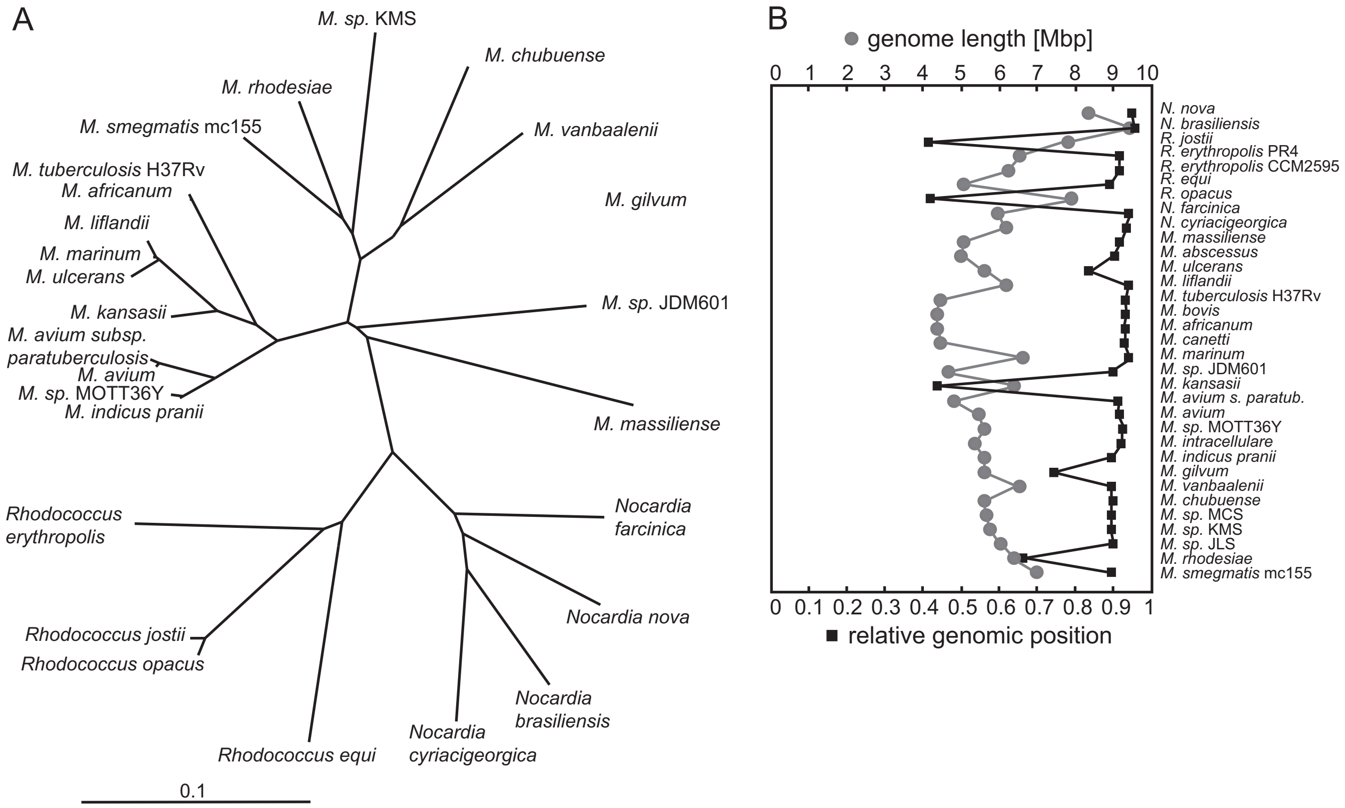

Supplementary Figure 2. Phylogenetic tree of Ms1 homologs and their positions in bacterial genomes. (A) The phylogenetic tree of Ms1 sRNAs created by the program ClustalW (1) is shown. **(B)** The position of Ms1 homologs in the genome is conserved in the majority of other species and is independent of genome length. The positions indicated with black squares show the relative position within the genome. The size of the genome is indicated with grey circles.

Supplementary Figure 3

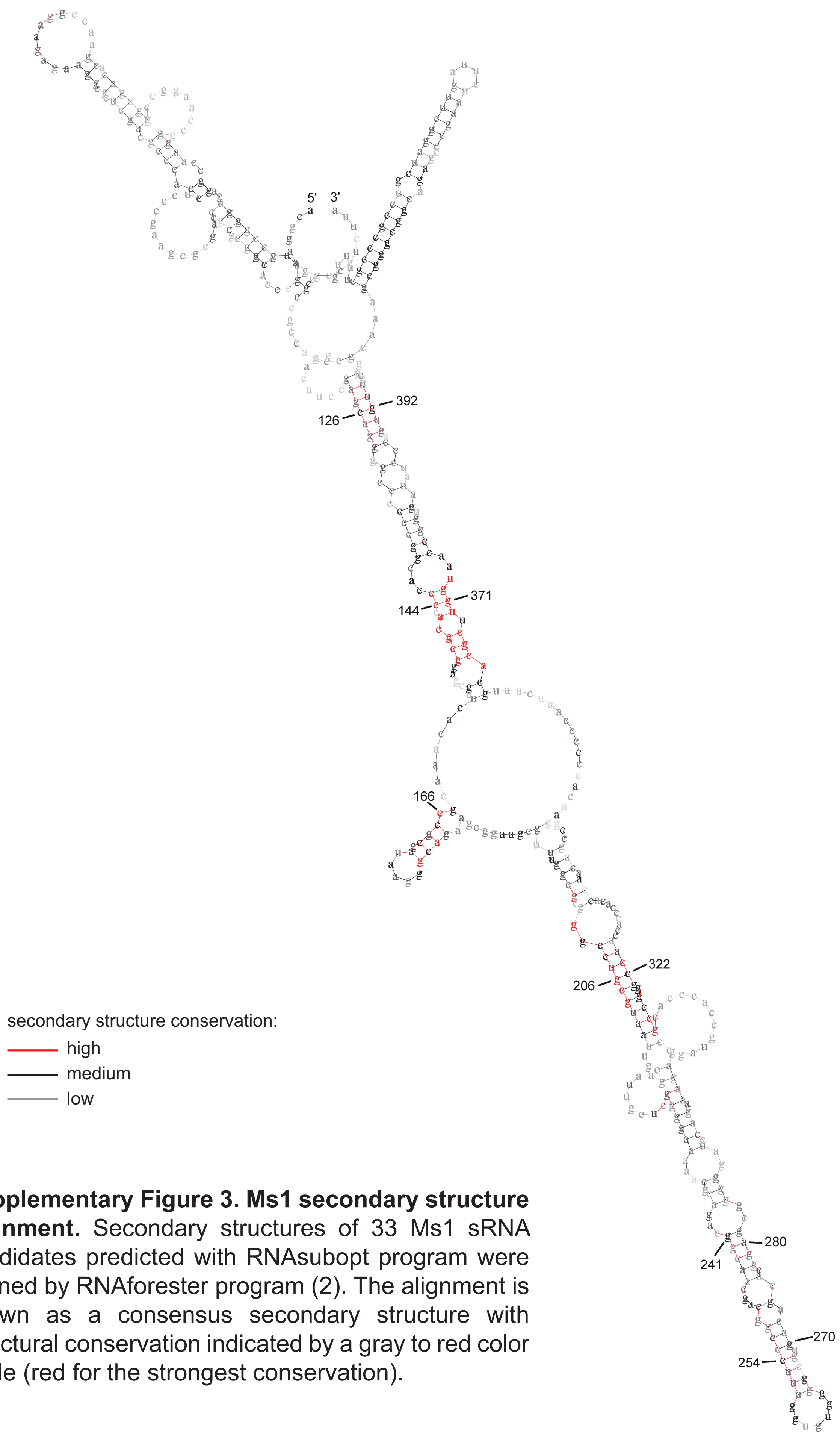

Supplementary Figure 3. Ms1 secondary structure alignment. Secondary structures of 33 Ms1 sRNA candidates predicted with RNAsubopt program were aligned by RNAforester program (2). The alignment is shown as a consensus secondary structure with structural conservation indicated by a gray to red color scale (red for the strongest conservation).

Supplementary Figure 4

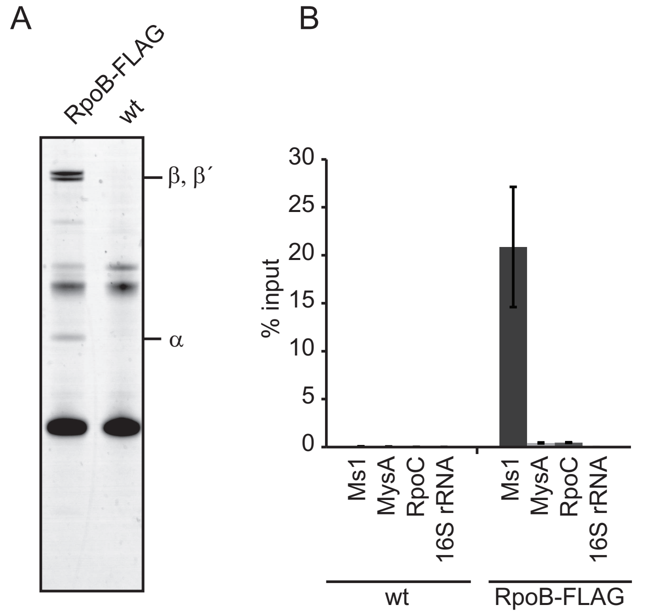

Supplementary Figure 4. **Interaction of Ms1 with RNAP containing a FLAG-tagged β subunit. (A)** RNAP subunits were pulled down from the strain carrying FLAG-tag RpoB (β subunit), but not from the wt control strain. **(B)** RNA that was pulled together with FLAG-tag RpoB was isolated and the presence of Ms1 quantified by RT-qPCR. Ms1 was pulled down in the strain with RpoB-FLAG, but not in the wt strain. In addition, no control mRNA - *mysA* (σ^A mRNA), *rpoC* (RNAP β´ subunit mRNA) nor 16S rRNA were pulled down in either the wt or RpoB-FLAG strain.

List of Primers

Ec – primers used to amplify sequences from *E. coli*; Ms - primers used to amplify sequences from *M. smegmatis*; the identity of the gene that was amplified with a particular primer is included in the name of the primer

Primers used for qPCR (5' to 3'):

Ms_Ms1_ F	GCCGGAAGAGAAGGCTAGAT
Ms_Ms1_ R	CGTCCGCTTTTCGAAACTAC
	these primers amplify both Ms1 and Ms1nb with similar efficiency
Ms_MysA_F	CCAAGGGCTACAAGTTCTCG
Ms_Mys_R	CTTGTTGATCACCTCGACCA
Ms_RpoC_F	CGACGAGATCTGGAACACCT
Ms_RpoC_R	GGTGAAGTACTCGCCGTAGC
Ms_16SrRNA_F	TCATGTTGCCAGCACGTTAT
Ms_16SrRNA_R	AAGGGGCATGATGACTTGAC
Ec_6SRNA_F	AGTCCCCTGAGCCGATATTT
Ec_6SRNA_ R	CGCAGTTTTAAGGCTTCTCG
Ec_RpoD_ F	TTCGTACGCAAGAACGTCTG
Ec_RpoD_ R	AGGTATCGCTGGTTTCGTTG
Ec_RpoC_F	GAAACCAACTCCGAAACCAA
Ec_RpoC_R	CGGTCAGGATCATCCACTCT
Ec_16SrRNA_F	CAGAAGAAGCACCGGCTAAC
Ec_16SrRNA_R	CGGGGATTTCACATCTGACT

Primers used to amplify template for T7 RNA polymerase *in vitro* transcription (5' to 3'):

Ms1_IVT_F	TACGTAATACGACTCACTATAGGAGAACGGAAGCTTGGCGAGGC
Ms1_IVT_R	AAAAGAAATCGGGCGGCGTCCCGGACAATGTCCGG

DNA Sequences used to create the *rrnB*-Ms1 and *rrnB*-Ms1nb fusions (5' to 3')

The DNA sequences listed below were synthesized by Invitrogen (GeneArt Strings DNA Fragments). The sequence highlighted in grey was not part of the commercially synthesized sequences and was added subsequently by PCR (the sequence is included in the Ms1_pJAM2_R primer – see next page).

rrnB ribosomal promoter   initiation nucleotide

GTGACCGCGTCTGACCAGGGAAAATAGCCCTCTGACCTGGGGATTTGACTCCCAGTTT
CCAAGGACGTAACTTATTCCAGG

Ms1nb

ACGGAAGCTTGGCGAGGCCAAGACCCAGCCGGAAGAGAAGGCTAGATCTCCCGACCC
AAGCTCCTAGCACGGATACCGAGCACCCACGCGGAGCGTTGCGGACCTGCGTAGTTTC
GAAAAGCGGACGGCCACGACGGCCCTTTGGGTGGGGTTGCAGCCGTAGCGCATCGCA
AAGACGCCGAGGTCACCCACGCAACGCACGCTTGGTCACTCGGGGTCCGTGCTAGCG
GGCGGCGAACCGGACATTGTCCGGGACGCCGCCCGATTTCTTTT

Ms1

ACGGAAGCTTGGCGAGGCCAAGACCCAGCCGGAAGAGAAGGCTAGATCTCCCGACCC
AAGCTCCTAGCACGGATACCGAGCACCCACGCGGAGCACATGCCGCGGAATAGGCAAA
AGTGTTGCGGACCTGCGTAGTTTCGAAAAGCGGACGGCCACGACGGCCCTTTGGGTGG
GGTTGCAGCCGTAGCGCATCGCAAAGACGCCGAGGTCACCCACGCAACCCACATCGCA
CGCTTGGTCACTCGGGGTCCGTGCTAGCGGGCGGCGAACCGGACATTGTCCGGGACG
CCGCCCGATTTCTTTT

The commercially obtained DNAs were then used as templates for PCR (Phusion High-Fidelity DNA Polymerase, New England Biolabs) with primers carrying ~20 nt overlaps (primers were designed according to manufacturer´s instructions for GeneArt Seamless Cloning and Assembly Kit, Invitrogen) in these combinations:

rrnB sequence:
pJAM2_rrnB80_F 5‘-ATAAAGCTAGTCACGGTGACCGCGTCTGACCAGGG-3´
rrnB_Ms1_R 5´-CCTCGCCAAGCTTCCGTCCTGGAATAAGTTACGTCCTTG-3´

Ms1 and Ms1nb sequence:
Ms1_F 5´-ACGGAAGCTTGGCGAGGC-3´
Ms1_pJAM2_R 5´-TGTCTAGAAGTACTGAAAAGAAATCGGGCGGCGTCCCGGACAATGTCCGG-3´

The obtained PCR products were purified by QIAquick PCR Purification Kit (Qiagen) and recombined together with linearized pJAM2 with deleted acetamidase promoter according to manufacturer´s instructions (GeneArt Seamless Cloning and Assembly Kit, Invitrogen) and transformed into One Shot TOP10 chemically Competent *E. coli.*

Enzymatic digestion, MALDI mass spectrometry and protein identification

Coomassie Brilliant Blue-stained protein spots were excised from the gel, cut into small pieces and destained using 50 mM 4-ethylmorpholine acetate (pH 8.1) in 50% acetonitrile (MeCN). The proteins were further reduced with 30 mM TCEP in 100 mM Tris-HCl pH 8.0 at 65°C for 30 min and alkylated with 30 mM iodacetamide in 100 mM Tris-HCl pH 8.0 for 60 min in the dark. The gel was washed with water, shrunk by dehydration in MeCN and reswelled again in water. The supernatant was removed and the gel was partly dried in a SpeedVac concentrator. The gel pieces were then incubated overnight at 37 °C in a cleavage buffer containing 25 mM 4-ethylmorpholine acetate, 5% MeCN and trypsin (100 ng; Promega). The resulting peptides were extracted to 40% MeCN/0.1% TFA. An aqueous 50% MeCN/0.1% TFA solution of α-cyano-4-hydroxycinnamic acid (5 mg/ml; Sigma Aldrich) was used as a MALDI matrix. One μl of the peptide mixture was deposited on the MALDI plate, allowed to air-dry at room temperature and overlaid with 0.4 μl of the matrix. Mass spectra were measured on an Ultraflex III MALDI-TOF instrument (Bruker Daltonics, Bremen, Germany) in the mass range of 700-4000 Da and calibrated internally using the monoisotopic $[M+H]^+$ ions of trypsin autoproteolytic fragments (842.5 and 2211.1 Da). The peak lists created using the flexAnalysis 3.3 program were searched using the in-house MASCOT search engine against the SwissProt 2013_09 or NCBInr 20130918 database subset of bacterial proteins with the following search settings: peptide tolerance of 30 ppm, missed cleavage site value set to one, variable carbamidomethylation of cysteine, oxidation of methionine and protein N-terminal acetylation. Proteins with a MOWSE score over the threshold of 56 (SwissProt) or 77 (NCBInr) calculated for the settings used were considered to have been identified. If the score was lower or only slightly higher than the threshold value, the identity of the protein candidate was confirmed by MS/MS analysis.

Supplementary Table 1. Genomic characterization of Ms1 homologs.

	E-value	5'	3'	genome length	5' relative	3' relative	strand	5' flank. gene	3' flank. gene
**Mycobacterium smegmatis str. MC2 155**	5,00E-153	6242368	6242671	6988209	0,89327	0,89331	+	morphological differentiation-associated protein	transcriptional regulator, IclR family protein
**Mycobacterium rhodesiae NBB3**	3,00E-87	2176136	2175834	6415739	0,33919	0,33914	-	?	?
**Mycobacterium sp. MCS**	1,00E-73	5092319	5092618	5705448	0,89254	0,89259	+	HAD-superfamily subfamily IB hydrolase, TIGR01490	hypothetical protein
**Mycobacterium sp. KMS**	1,00E-73	5129977	5130276	5737227	0,89416	0,89421	+	HAD-superfamily subfamily IB hydrolase, TIGR01490	hypothetical protein
**Mycobacterium sp. JLS**	1,00E-73	5444334	5444633	6048425	0,90012	0,90017	+	HAD-superfamily subfamily IB hydrolase, TIGR01490	hypothetical protein
**Mycobacterium chubuense NBB4**	3,00E-73	5021204	5021510	5583723	0,89926	0,89931	+	HAD-superfamily subfamily IB hydrolase, TIGR01490	hypothetical protein
**Mycobacterium vanbaalenii PYR-1**	5,00E-65	5804185	5804455	6491865	0,89407	0,89411	+	?	?
**Mycobacterium gilvum PYR-GCK**	7,00E-63	1437598	1437327	5619607	0,25582	0,25577	-	hypothetical protein	HAD-superfamily subfamily IB hydrolase, TIGR01490
**Mycobacterium intracellulare ATCC 13950**	8,00E-56	421306	421017	5402402	0,077985	0,077931	-	hypothetical protein	HAD-superfamily subfamily IB hydrolase, TIGR01490
**Mycobacterium indicus pranii MTCC 9506**	8,00E-56	595492	595203	5589007	0,10655	0,1065	-	hypothetical protein	HAD-superfamily subfamily IB hydrolase, TIGR01490
**Mycobacterium sp. MOTT36Y**	1,00E-54	415046	414757	5613626	0,073935	0,073884	-	hypothetical protein	HAD-superfamily subfamily IB hydrolase, TIGR01490
**Mycobacterium avium 104**	2,00E-51	458797	458507	5475491	0,083791	0,083738	-	hypothetical protein	HAD-superfamily subfamily IB hydrolase, TIGR01490
**Mycobacterium avium subsp. paratuberculosis str. k10**	2,00E-51	438510	438220	4829781	0,090793	0,090733	-	hypothetical protein	hypothetical protein
**Mycobacterium kansasii ATCC 12478**	3,00E-48	2811382	2811648	6432277	0,43707	0,43712	-	Inhibition of morphological differentiation protein	hypothetical protein
**Mycobacterium sp. JDM601**	1,00E-47	4192229	4192522	4643668	0,90278	0,90285	+	phosphoserine phosphatase SerB	hypothetical protein
**Mycobacterium marinum M**	4,00E-47	6233431	6233734	6636827	0,93922	0,93926	+	?	?
**Mycobacterium africanum GM041182**	5,00E-46	4076624	4076920	4389314	0,92876	0,92883	+	hypothetical protein	hypothetical protein
**Mycobacterium canettii CIPT 140010059**	5,00E-46	4170632	4170928	4482059	0,93052	0,93058	+	hypothetical protein	hypothetical protein

**Mycobacterium bovis BCG str. Tokyo 172 DNA**	5,00E-46	4071048	4071344	4371711	0,93123	0,93129	+	hypothetical protein	hypothetical protein
**Mycobacterium tuberculosis H37Rv**	6,00E-45	4100862	4101158	4411709	0,92954	0,92961	+	HAD-superfamily subfamily IB hydrolase, TIGR01490	hypothetical protein
**Mycobacterium ulcerans Agy99**	2,00E-44	4707988	4708291	5631606	0,83599	0,83605	+	?	?
**Mycobacterium liflandii 128FXT**	2,00E-44	5839498	5839801	6208955	0,9405	0,94054	+	?	?
**Nocardia nova SH22a**	1,00E-32	431354	431076	8348532	0,051668	0,051635	-	?	?
**Nocardia brasiliensis ATCC 700358**	2,00E-31	429932	429652	9436348	0,045561	0,045532	-	hypothetical protein	putative hydrolase
**Mycobacterium massiliense str. GO 06**	8,00E-31	432566	432303	5068807	0,085339	0,085287	-	Glutathione import ATP-binding protein GsiA	Uncharacterized protein
**Mycobacterium abscessus subsp. bolletii 50594**	8,00E-31	486041	485778	5000473	0,097199	0,097146	-	hypothetical protein	hypothetical protein
**Nocardia cyriacigeorgica GUH-2 chromosome**	2,00E-20	383868	383586	6194645	0,061968	0,061922	-	conserved protein of unknown function	putative hydrolase
**Nocardia farcinica IFM 10152 DNA**	8,00E-18	367388	367133	6021225	0,061015	0,060973	-	hypothetical protein	hypothetical protein
**Rhodococcus opacus B4 DNA**	1,00E-15	4576105	4575820	7913450	0,57827	0,57823	-	hypothetical protein	hypothetical protein
**Rhodococcus erythropolis CCM2595**	8,00E-12	526261	526022	6281198	0,083784	0,083745	-	hypothetical protein	hypothetical protein
**Rhodococcus erythropolis PR4 DNA**	8,00E-12	556109	555870	6516310	0,085341	0,085304	-	hypothetical protein	hypothetical protein
**Rhodococcus jostii RHA1**	3,00E-11	4570369	4570084	7804765	0,58559	0,58555	-	NaN	NaN
**Rhodococcus equi 103S chromosome**	3,00E-11	560162	559909	5043170	0,11107	0,11102	-	hypothetical protein	putative haloacid dehalogenase-like hydrolase

E-value - BLAST Expect value of the listed sequences relative to the *Mycobacterium smegmatis* Ms1. E-value is a statistical parameter, which characterizes the significance of an alignment. The lower the E-value, the lower the probability that the alignment is a random result.

5‘, 3‘ – first and last nucleotides of the aligned sequences

Genome length – the length of the relevant genome in bp

5‘, 3‘ relative – relative position within the genome whose size (starting at ori) was taken as 1

Strand – orientation of the strand (either + or -)

5‘, 3‘ flank. gene – genes flanking Ms1 homologs, ? – no annotated ORF exists for these regions in the database

The Ms1 sRNA candidates were detected by BLASTing (3) the *M. smegmatis* Ms1 sequence against NCBI nr collection using the default parameters except of Word size that was set to 7 to obtain even less similar sequences. The obtained hits could be divided into two classes according to E-value. The first class contained hits with E-value < 10^{-11} and the corresponding sequences were considered to be Ms1

candidates. Then, there was a large gap in E-value where we did not obtain any hits (from 10^{-11} to 10^{-3}). The second class contained hits with E-value > 10^{-3} and these were considered unlikely to be Ms1 homologs. The sequences of the Ms1 candidates were then retrieved from the NCBI bacterial genome collection according to BLAST hits genomic loci.

Supplementary Table 2. Mass spectrometric identification of *M. smegmatis* abundant proteins in Ms1 glycerol gradient fractions.

Accession number	Protein	No. pept.	SC [%]	MS/MS analysis
**MSMEG_1368**	rpoC, DNA-directed RNA polymerase subunit beta'	11	10	TFHQGGVTGGADIVGGLPR<br>VTIIDSGSTEFLPGSLTER
**MSMEG_1367**	rpoB, DNA-directed RNA polymerase subunit beta	12	11	VNPFGFIETPYR
**MSMEG_5049**	sucA, Multifunctional 2-oxoglutarate metabolism enzyme	14	11	IGDAHLALPEGFTVHPR
**MSMEG_4954**	transcription termination factor Rho	8	15	AGILDVLDNYAFVR
**MSMEG_1807**	acetyl-/propionyl-coenzyme A carboxylase subunit alpha	15	29	SVAVYAEPDADAPHVR<br>VSLPGDLAIGGGGGAAAPGVVR
**MSMEG_1813**	propionyl-CoA carboxylase subunit beta	6	15	GYVDAVIPPSHTR<br>AGYAGNIVVGFGR<br>LQQTYEDTLVNPYIAAER
**MSMEG_4290**	GlnA1, Glutamine synthetase 1	7	21	SVFEDGLAFDGSSVR<br>LVPGYEAPINLVYSQR<br>SGWWNTGEPFESDGSANR
**MSMEG_6242**	alcohol dehydrogenase	5	12	LAQDVGIPDNFSQVR<br>FHPFPR
**MSMEG_1524**	rpoA, DNA-directed RNA polymerase subunit alpha	9	28	SFALPIDDLDLTVR
**MSMEG_1878**	S30AE family protein, RafS	4	13	NVEVPDHFR<br>HAFDYGLIR

No. pept. = Number of matched peptides, SC = Sequence coverage in %

Supplementary Table 3. Mass spectrometric identification of *M. smegmatis* proteins interacting with *in vitro* transcribed Ms1 RNA.

Accession number	Protein	No. pept.	SC [%]	MS/MS analysis
**MSMEG_2656**	Polyribonucleotide nucleotidyltransferase, guanosine pentaphosphate synthetase I, gpsI	10	20	AVFDMVVAGR TTDFGAFVSLLPGR ALVPVLPSIEEFPYAIR IALIDGTWVAFPTVEQLER
**MSMEG_4954**	transcription termination factor Rho	10	23	VFPAVDVNPSGTR TTILQDIANAITR VLSGLDSHQAIDLLISQLR TAEASSPATADAQTGEQAAEAPAPR

No. pept. = Number of matched peptides, SC = Sequence coverage in %

Supplementary Table 4. Mass spectrometric identification of proteins interacting with *E.coli* RNA polymerase imunoprecipitated by the 8RB13 antibody.

Protein	No. pept.	SC [%]	MS/MS analysis
CbpA**, Curved DNA-binding protein**	16	56	FKEVAEAWEVLSDEQR ESILLTIPPGSQAGQR
NusG**, Transcription antitermination protein NusG**	10	55	WYVVQAFSGFEGR

No. pept. = Number of matched peptides, SC = Sequence coverage in %

List of strains and plasmids

Strain/plasmid	Relevant characteristics	Source
Bacillus subtilis		
168	wt	Laboratory strain
Escherichia coli		
K12 KW72	wt	(4), Tamas Gaal, University of Wisconsin-Madison, USA
LK1270	pJAM2 *kan*	(5)
LK1282	pJAM2*mysA kan*	(6)
LK1314	pJAM2-*rrnB*-Ms1nb *kan*	This study
LK1315	pJAM2-*rrnB*-Ms1 *kan*	This study
Mycobacterium smegmatis		
mc^2155	wt	Laboratory strain
MR-sspB	*rpoB*-FLAG *hyg kan*	(7)
LK1302	mc^2 155 pJAM2 *kan*	This study
LK1304	mc^2 155 pJAM2*mysA kan*	This study
LK1323	mc^2 155/pJAM2-*rrnB*-Ms1 *kan*	This study
LK1337	mc^2 155/pJAM2-*rrnB*-Ms1nb *kan*	This study

References

1. Thompson, J.D., Higgins, D.G. and Gibson, T.J. (1994) CLUSTAL W: improving the sensitivity of progressive multiple sequence alignment through sequence weighting, position-specific gap penalties and weight matrix choice. *Nucleic Acids Res*, **22**, 4673-4680.
2. Hofacker, I.L. (2004) RNA secondary structure analysis using the Vienna RNA package. *Curr Protoc Bioinformatics*, **Chapter 12**, Unit 12.12.
3. Altschul, S.F., Gish, W., Miller, W., Myers, E.W. and Lipman, D.J. (1990) Basic local alignment search tool. *J Mol Biol*, **215**, 403-410.
4. Trotochaud, A.E. and Wassarman, K.M. (2004) 6S RNA function enhances long-term cell survival. *J Bacteriol*, **186**, 4978-4985.
5. Triccas, J.A., Parish, T., Britton, W.J. and Gicquel, B. (1998) An inducible expression system permitting the efficient purification of a recombinant antigen from Mycobacterium smegmatis. *FEMS Microbiol Lett*, **167**, 151-156.
6. China, A. and Nagaraja, V. (2010) Purification of RNA polymerase from mycobacteria for optimized promoter-polymerase interactions. *Protein Expr Purif*, **69**, 235-242.
7. Kim, J.H., Wei, J.R., Wallach, J.B., Robbins, R.S., Rubin, E.J. and Schnappinger, D. (2011) Protein inactivation in mycobacteria by controlled proteolysis and its application to deplete the beta subunit of RNA polymerase. *Nucleic Acids Res*, **39**, 2210-2220.
